# The extracellular space and epileptic activity in the adult brain: Explaining the antiepileptic effects of furosemide and bumetanide

**DOI:** 10.1111/j.1528-1167.2012.03471.x

**Published:** 2012-05-21

**Authors:** Daryl W Hochman

**Affiliations:** Departments of Surgery (Surgical Sciences) and Pharmacology and Cancer Biology, Duke UniversityDurham, North Carolina, U.S.A.

**Keywords:** Extracellular space, Furosemide, Bumetanide, Epilepsy, Astrocytes, Intrinsic optical signal, NKCC1, KCC2

## Abstract

Treatments that modulate the size of the extracellular space (ECS) also block epileptiform activity in adult brain tissue. This includes the loop diuretics furosemide and bumetanide, and alterations of the osmolarity of the ECS. These treatments block epileptiform activity in a variety of laboratory adult seizure models regardless of the underlying synaptic and physiologic mechanisms generating the seizure activity. Optical imaging studies on adult hippocampal slices show that the blockade of epileptiform activity by these treatments is concomitant with their blockade of activity-driven changes of the ECS. Here we develop and analyze the hypothesis that activity-driven changes in the size of the ECS are *necessary* for the maintenance of hypersynchronous epileptiform activity. In support of this hypothesis is an accumulation of data from a number of studies suggesting that furosemide and bumetanide mediate antiepileptic effects through their blockade of cell swelling, dependent on their antagonism of the glial Na+-K-2Cl cotransporter (NKCC1).

It has long been hypothesized that volume and ion changes in the extracellular space (ECS) can modulate the excitability and epileptogenicity of tissue (Andrew, 1991; Jefferys, 1995; Dudek et al., 1998). Neuronal networks interact with the surrounding ECS in a dynamic, feedback-loop manner. Action potential firing can change the ion concentrations and volume of the ECS, and likewise these changes in the ECS are thought to modulate synaptic transmission and neuronal excitability (Hochman, 2009). The proportion of a volume of brain tissue that is composed of the ECS is called the extracellular volume fraction (EVF). The EVF is a dynamic entity that can change within localized microscopic regions in response to neuronal activity. Action potential firing and synaptic activity generate localized increases in extracellular potassium and chloride. These ion gradients are dispersed, in part, via movement into glial cells through membrane-bound ion transporters and channels (Sontheimer, 1994; Chen & Nicholson, 2000; Emmi et al., 2000; Simard & Nedergaard, 2004). These changing ion concentrations generate osmotic gradients between extracellular and intracellular compartments, causing the diffusion of water into hypertonic spaces. The end result is an activity-driven movement of water from intracellular compartments into glial cells, mediating a transient reduction of the EVF through glial cell swelling (Simard & Nedergaard, 2004; Østby et al., 2009). These considerations suggest that the microscopic organization of glial cell processes could potentially contribute significantly to the ionic and volume changes of the ECS. An electron microscopy study showed that glial cell processes proliferate within specific microdomains in response to increases in neuronal activity during the induction of long-term potentiation (LTP) (Wenzel et al., 1991). It may be that epileptiform activity also alters the distribution of astrocytic processes in ways that are important in epileptogenesis.

We developed the following hypothesis: activity-driven changes in the EVF are *necessary* for the maintenance of hypersynchronized epileptiform activity in adult brain tissue. This is not to deny the necessity of synaptic mechanisms for the generation and maintenance of epileptic activity, and their importance as targets for the development of antiepileptic drugs. Rather, our hypothesis is that regardless of the specific synaptic mechanisms underlying the generation of a particular type of seizure model, it is always possible to disrupt that activity by blocking the changes in the EVF that result from neuronal discharge. As such, this particular type of nonsynaptic mechanism is also a target deserving serious consideration for development of new therapeutics. Results to support this notion are considered from experiments in which either one of two types of the following treatments were studied in laboratory seizure models: (1) modulation of cation-chloride transport, either with manipulation of extracellular ions or loop diuretics (e.g., furosemide and bumetanide); and (2) modulation of the osmolarity of the ECS. Two assumptions are made in what follows. The first assumption is that “hypersynchronized” epileptiform activity is identified with nearly simultaneous (i.e., hypersynchronous) discharging of action potentials from populations of neurons. This excludes phenomena such as neuronal or synaptic hyperexcitability without hypersynchrony, as well as spontaneously occurring action potentials from individual neurons that do not occur as part of a hypersynchronized population discharge.

The second assumption is that if a particular treatment blocks epileptiform activity in a number of different seizure models, there is a set of common mechanisms through which that treatment blocks epileptiform activity in each of the models. For example, it has been shown that furosemide blocks epileptiform activity in the nonsynaptic “zero-calcium” model, as well is in a variety of other models where the epileptiform activity is dependent on different types of synaptic mechanisms in each of the models. In view of these results, the common mechanism (or set of mechanisms) through which furosemide blocks epileptiform activity across all models must be nonsynaptic. This does not exclude the possibility that a treatment might also modulate epileptogenicity through mechanisms that are unique to some specific model, in *addition* to its antiepileptic mechanisms that are common across all models. As an example, in a model of high-frequency stimulation, it is likely that furosemide mediates an antiepileptic effect through its blockade of the neuron-specific K+Cl− cotransporter (KCC2) (Viitanen et al., 2010). We suggest that even in such cases, furosemide also exerts antiepileptic effects through its other nonsynaptic mechanisms that are common to all models that, in principle, would be sufficient to block seizure discharge independent of its additional model-specific effects. This second assumption is motivated by a desire to propose the simplest general mechanism that can explain a large body of results. Future studies might find that furosemide (or any of the other treatments discussed) mediates its antiepileptic effects through a spectrum of different mechanisms, and its ability to block epileptiform activity in any one seizure model might depend on mechanisms that are distinct and entirely independent from its antiepileptic mechanisms in other models. Our only claim here is that we are able to propose a reasonable hypothesis requiring a single mechanism that is consistent with currently available experimental results.

## KCC2 and NKCC1

Many of the experiments discussed here used treatments that are known to modulate ion cotransporters on neurons and glia, particularly a neuronal isoform of the KCC2 and the Na+-K-2Cl cotransporter (NKCC1) that is present on both neurons and glia (Russel, 2000; Blaesse et al., 2009). Under normal physiologic conditions, KCC2 transports K+ and Cl− from the intracellular spaces of neurons into the ECS, and NKCC1 transports Na+, K+, and Cl− from the ECS into the intracellular spaces of neurons and glia. The loop diuretics, furosemide (Lasix) and bumetanide (Bumex) are classic NKCC1 antagonists, with bumetanide being a more potent and specific antagonist than furosemide (Russel, 2000). Reduction of extracellular chloride (low-[Cl^−^]_o_) by equimolar substitution with impermeant anions such as gluconate, also antagonizes NKCC1. Furosemide antagonizes KCC2 in addition to NKCC1, and can thus reduce γ-aminobutyric acid receptor A (GABA_A_) inhibition in adult neurons by reducing the neuronal transmembrane chloride gradient (Thompson et al., 1988). Both furosemide and low-[Cl^−^]_o_ treatments have been shown to block activity-driven glial cell swelling (Kimelberg & Frangakis, 1985; Ransom et al., 1985; Walz & Hinks, 1985).

## Results from Intrinsic Optical Signal Imaging Studies

**Figure 1:**
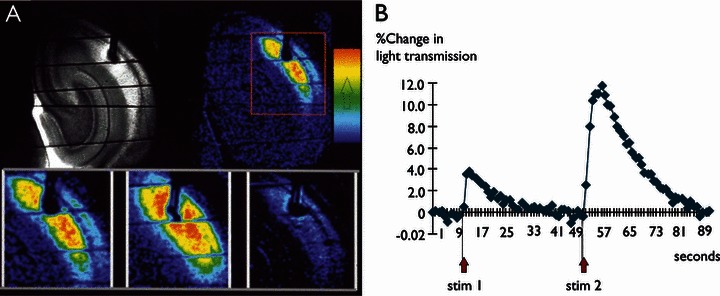
Imaging of intrinsic optical signal in tissue slices. (**A**) Top left panel: Grayscale image of a hippocampal slice with a stimulating electrode placed on the Schaffer collateral. Top right panel: Pseudo-colored image of the intrinsic signal evoked by 2 s of electrical stimulation. Red represents a 12% change, and dark-blue is 0%. The largest optical changes occur in the dendritic layers. Lower panels: The area from the upper right panel, surrounded by a red box, is shown magnified in the lower panels. The leftmost panel shows the optical response to a smaller stimulation current, compared to the middle panel that was stimulated using current at three times the amplitude. The bottom rightmost panel shows the optical response to a larger electrical stimulus 25 min after furosemide (2.5 mm) had been added to the perfusion medium. (**B**) A plot of the %-change in light transmission versus time (seconds). The two stimuli used to generate the optical images shown in (**A**) (lower left and lower middle panels) are plotted to show the quantitative changes in time. Note that although the each of the two stimuli lasted for only 2 s, the recovery (which presumably represents the recovery of cell volume changes) lasted for tens of seconds.

Intrinsic optical signal (IOS) in brain tissue slices is thought to depend on light scattering changes that arise from the changing membrane geometry of cellular processes as they swell or shrink during increases or decreases in neuronal activity (MacVicar & Hochman, 1991). The IOS-imaging technique involves shining light through a tissue slice, and quantifying changes in light scattering from sequences of images acquired with a digital camera. For example, IOS experiments with hippocampal slices might involve measuring the synaptically evoked optical responses to Schaffer collateral stimulation during different pharmacologic treatments (Fig. 1). Using ion-selective microelectrodes, quantitative measurements of the EVF and extracellular potassium were measured simultaneously during IOS imaging of neocortical slices (Holthoff & Witte, 1996). This study showed that the magnitude of the synaptically evoked IOS varied linearly with the EVF, but the IOS was not well correlated to extracellular potassium. Treatment of the slices with either furosemide, or equimolar replacement of extracellular chloride with gluconate (low-[Cl^−^]_o_), similarly abolished both the optical signal and the EVF changes. However, the magnitude of the stimulation-evoked extracellular potassium changes were not affected equally by both of these treatments (20% reduction by low-[Cl^−^]_o_; 40% reduction by furosemide). Neither treatment abolished the activity-driven potassium changes, but they did abolish the activity-driven changes in IOS and EVF, suggesting that IOS imaging correlates well with the EVF in the neocortical slice. Furosemide and low-[Cl^−^]_o_ were also shown to abolish the intrinsic signal (and hence activity-driven changes of the EVF) in earlier experiments on rat hippocampal slices (MacVicar & Hochman, 1991). Neither furosemide nor low-[Cl^–^]_o_ appeared to reduce action potential generation or synaptically evoked field responses in hippocampal or neocortical slices.

Studies using the rat optic nerve have shown that repetitive activity causes a reduction of the EVF due to cell swelling, and that these activity-driven EVF changes occur only when astrocytes have proliferated and differentiated postnatally (Ransom et al., 1985). IOS imaging on the optic nerve showed that activity-driven optical changes could be elicited, and that increasing extracellular potassium alone could mimic their activity-evoked changes (MacVicar et al., 2002). In enucleated nerves (which have only astrocytes, and do not have any axons), increasing extracellular potassium induced optical changes that could be blocked by furosemide and bumetanide. Immunofluorescence and Western blot data showed the presence of NKCC1 in the optic nerve, localized to astrocytes.

Manipulations of the osmolarity of the ECS were also shown to mimic activity-driven changes in the EVF and IOS in hippocampal slices (Andrew & MacVicar, 1994). Decreasing the osmolarity of the ECS (i.e., diluting with water) caused cell swelling, a reduction of the EVF, and an increase in IOS. Increasing the osmolarity of the ECS, with the physiologically inert and impermeant osmolyte mannitol, elicited the opposite changes. It is notable that reducing the osmolarity increased the CA1 field responses to orthodromic stimulation, and increasing the osmolarity reduced the field responses, with the onset of these changes in excitability occurring at the same time as the onset of the changes in the IOS.

Finally, studies of the effects of furosemide on epileptiform activity in hippocampal slices have been performed in combination with IOS imaging (Hochman et al., 1995). It was observed that furosemide blocked epileptiform activity in a variety of slice seizure models. Action potential generation and excitatory synaptic drive were not diminished by furosemide treatment. The furosemide blockade of epileptiform activity was seen to occur concomitantly with the blockade of the activity-driven IOS.

Taken together, the results of this section support the following conclusions:

1. Furosemide and low-[Cl^−^]_o_ block activity-driven decreases in the EVF, likely by blocking glial cell swelling through antagonism of NKCC1.
2. Treatments that block the activity-driven changes in EVF also block epileptiform activity.

## Effects of Chloride-Cotransporter Antagonists on Epileptiform Activity

### General results

Furosemide has been shown to block epileptiform activity in many standard laboratory seizure models tested. In rat hippocampal slices, these include (1) afterdischarge activity in CA1 elicited by tetanic Schaffer collateral stimulation, high potassium (high-K^+^) (10 mm), both acute and prolonged bathing of slices in zero-magnesium medium, 4-aminopyridine (4-AP) (300 μm), bicuculline (100 μm), and zero-calcium (0-Ca+) (Hochman et al., 1995; Gutschmidt et al., 1999). Whole animal studies in rats showed that furosemide blocks kainic acid status in rats (Hochman et al., 1995; Schwartzkroin et al., 1998) and prevented sound-triggered seizures in audiogenic seizure-prone animals (Reid et al., 2000). Furosemide has also been shown to have antiepileptic effects in several studies on human subjects. Intravenously administered furosemide blocked spontaneously occurring interictal spiking and stimulation-evoked afterdischarges of the neocortex during intraoperative studies in patients with medically intractable seizures (Haglund & Hochman, 2005). In those studies, furosemide elicited profound antiepileptic effects on each subject regardless of their specific seizure type. A small clinical trial showed that furosemide significantly reduced seizure frequency in adults with refractory epilepsy (Ahmad et al., 1976).

Bumetanide, a more potent and specific antagonist of NKCC1 than furosemide, has also been studied in models of animal seizures. Bumetanide was found to be more potent than furosemide in blocking kainic acid–induced status in rats (Schwartzkroin et al., 1998), and in preventing sound-triggered seizures in audiogenic seizure-prone rats (Reid et al., 2000). Bumetanide was also found to be more potent than furosemide in blocking epileptiform activity generated by focal application of bicuculline or 4-AP to the primate cortex, as well as in blocking stimulation-evoked afterdischarges in primate cortex (Haglund & Hochman, 2009). However, results of bumetanide in slice experiments have not been as conclusive as those in in vivo studies (Margineanu & Klitgaard, 2006), an issue we will address in subsequent text.

Taken together, these results support the following conclusions:

1. Furosemide blocks epileptiform activity regardless of the underlying synaptic mechanisms generating the epileptiform activity.
2. Because furosemide blocks epileptiform activity elicited by the GABA_A_ antagonist bicuculline, it mediates its antiepileptic effects independent of GABA_A_-dependent signaling.
3. Furosemide blocks epileptiform activity elicited in the 0-Calcium model in which synaptic activity is absent. Hence the antiepileptic mechanisms of furosemide are nonsynaptic.
4. Because bumetanide more potently blocks epileptiform activity than furosemide in in vivo seizure models, and bumetanide is a more specific and potent antagonist of NKCC1 than is furosemide, antagonism of NKCC1 is the likely mechanism through which furosemide and bumetanide mediate their antiepileptic effects in seizure models in vivo.

### Hippocampal slice studies with gluconate replacement of extracellular chloride

Under normal physiologic conditions, NKCC1 transports Na+, K+, and Cl− from the extracellular to intracellular compartments. If antagonism of NKCC1 is a critical mechanism of the antiepileptic effects of loop diuretics, then reduction of extracellular chloride, which would reduce the efficiency of NKCC1, should also be antiepileptic. This notion was confirmed by a series of studies comparing the effects of furosemide to gluconate-replacement (low-[Cl^–^]_o_) in hippocampal slices, showing that both treatments elicited nearly identical antiepileptic effects on all slice seizure models tested (Hochman et al., 1999). Note that a reduction of extracellular chloride would increase (rather than decrease) the efficiency of the KCC1 neuronal cotransporter, which normally transports chloride from intracellular to extracellular spaces against its ion gradient. This is the opposite effect of furosemide, which antagonizes KCC1 (Geck & Pfeiffer, 1985; Cala, 1990). This represents further support for a critical role for NKCC1 antagonism in the antiepileptic effects of these treatments.

Because both furosemide and the reduction of extracellular chloride appear to elicit similar antiepileptic effects in slices, both similarly block activity-driven changes of the EVF (see optical imaging studies above), and both likely mediate their antiepileptic effects through antagonism of NKCC1, studies on the effects of either treatment alone can be reasonably interpreted as speaking to their common antiepileptic mechanisms.

When hippocampal slices were bathed in low-[Cl^−^]_o_ (7 mm) medium, intracellular sharp electrode recordings from CA1 and CA3 pyramidal cells showed that the ability of neurons to fire action potentials was not impaired in any detectable way, even after several hours of exposure to low-[Cl^−^]_o_ (Hochman & Schwartzkroin, 2000). Intracellular recordings during 4-AP bursting showed that after low-[Cl^–^]_o_ blocked the epileptiform burst activity, the large 4-AP–elicited spontaneous synaptic events remained undiminished and could still be recorded at the cell body. These results suggest that low-[Cl^−^]_o_ does not affect either synaptic transmission or transmission of synaptic input from the dendrite to the soma. In experiments in which all synaptic activity was blocked with transmitter receptor antagonists, measurements of the antidromic responses in the CA3 cell body layer to Schaffer collateral stimulation remained unaffected, even after several hours of treatment with low-[Cl^−^]_o_. This result suggests that the antiepileptic effects of low-[Cl^−^]_o_ are unlikely to involve effects on action potential generation, or effects of action potential propagation by major axonal branches.

Once epileptiform activity has been blocked completely in hippocampal slices, with either furosemide or low-[Cl^−^]_o_, intracellular recordings from CA1 pyramidal cells during Schaffer collateral stimulation show that neurons continue to respond to excitatory synaptic drive with hyperexcited responses (i.e., electrical stimuli applied to the Schaffer collaterals, which normally elicit single action potentials from neurons before furosemide or low-[Cl^−^]_o_ treatment, will elicit multiple action potentials or bursts of action potentials from neurons during treatment) (Hochman et al., 1999; Hochman & Schwartzkroin, 2000). This observation suggests that NKCC1 antagonism does not diminish excitatory synaptic drive or neuronal hyperexcitability at the time during which it completely blocks hypersynchronous neuronal discharge.

After prolonged treatment of hippocampal slices with furosemide or low-[Cl^−^]_o_, CA1 and CA3 field responses to Schaffer collateral stimulation are completely abolished in the cell body layers (Hochman & Schwartzkroin, 2000). Indeed, this observation has led some to conclude that furosemide may be poisoning the tissue (Gutschmidt et al., 1999). However, intracellular recordings from pyramidal cells during the blockade of field responses, as described in the previous paragraph, show that this is not the case. Instead, as revealed by pairs of intracellular recordings simultaneously recorded from two closely situated CA1 pyramidal cells, prolonged antagonism of NKCC1 desynchronizes the timing of their action potentials (Hochman & Schwartzkroin, 2000). Initially, prior to treatment with furosemide or low-[Cl^–^]_o_, action potentials simultaneously recorded from two CA1 pyramidal cells occur at almost the same instant, whether in response to Schaffer collateral stimulation or are when firing together during a hypersynchronized epileptiform discharge. After exposure to furosemide or low-[Cl^−^]_o_, the CA1 field response begins to diminish in amplitude and increases in breadth, as the synchronization between pairs of pyramidal cells begins to be lost. The field response is completely lost when pairs of action potentials become sufficiently desynchronized.

In summary, the results of slice experiments using furosemide and low-[Cl^−^]_o_ treatments support the following conclusions:

1. Both furosemide and low-[Cl^−^]_o_ mediate their antiepileptic effects through antagonism of NKCC1.
2. NKCC1 antagonism does not affect action potential generation or diminish excitability of presynaptic or postsynaptic neurons.
3. NKCC1 antagonism does not affect conduction of action potentials by presynaptic axons or the release of transmitter by presynaptic terminals.
4. NKCC1 antagonism does not affect the conduction of synaptic potentials from the dendrites to the soma of postsynaptic neurons.
5. The critical antiepileptic effect of NKCC1 antagonism appears to be the desynchronization of the firing of action potentials between pairs of neurons that otherwise fire hypersynchronously. A critical mechanism in the antiepileptic effects of furosemide and low-[Cl^−^]_o_ is likely to be their desynchronizing effects.

## Data from Experiments in Which the Osmolarity of the ECS Is Modulated

If the antiepileptic (desynchronizing) effects of furosemide and low-[Cl^−^]_o_ depend on their abolishment of activity-driven volume changes of the EVF (as a consequence of their antagonism of NKCC1), then manipulation of the EVF through other treatments should also affect epileptiform activity. Indeed, it has been long known in the clinical literature that increasing plasma osmolarity reduces ongoing seizure activity and the likelihood of having seizure, whereas decreasing plasma osmolarity has the opposite effect (Andrew, 1991). Intraoperative studies on humans with refractory epilepsy showed that mannitol (50 g intravenous bolus injection) blocked spontaneous interictal spiking and electrical stimulation evoked afterdischarge activity of the neocortex as effectively as furosemide (20 mg intravenous bolus injection) (Haglund & Hochman, 2005).

The antiepileptic effects of increased extracellular osmolarity in humans have been reproduced in a variety of laboratory seizure models. In vivo studies in rats showed that systemically administered hyperosmotic solutions increase electroshock seizure thresholds (Reed & Woodbury, 1964) and prevent the development of kainic acid–induced seizures (Baran et al., 1987). In vitro studies showed the increasing osmolarity in hippocampal slices during 0-Ca^2+^ reduced or blocked the nonsynaptically mediated synchronized discharges, whereas decreasing osmolarity had the opposite effect (Dudek et al., 1990; Roper et al., 1992). Studies on hippocampal slices bathed in high-potassium medium showed that changes in osmolarity could suppress the epileptiform activity, and that alteration of the size of the EVF is a critical component in the generation of epileptiform activity (Traynelis & Dingledine, 1989).

To summarize, the main conclusion supported by these studies is that increasing osmolarity of the ECS simultaneously increases the EVF and suppresses epileptic activity, both in synaptic and nonsynaptic seizure models. Decreasing the osmolarity of the ECS does the inverse, reducing the EVF and increasing epileptogenicity in seizure models.

## The Extracellular Space Is Necessary for Furosemide to Suppress Epileptiform Activity

When furosemide is applied to synaptically coupled excitatory and inhibitory neurons in culture, it elicits hypersynchronized bursting (Jarolimek et al., 1996). Presumably, this proepileptic effect of furosemide is mediated by its antagonism of KCC2, and the consequent reduction of GABA_A_-mediated currents. That is, without the structure of the extracellular space provided by the glial cells, and without its action on NKCC1-mediated glial cell swelling, furosemide elicits, rather than blocks, epileptiform activity.

## Data Possibly Contrary to Our Hypothesis

There is evidence that bumetanide elicits antiepileptic effects in neonate seizure models through synaptic mechanisms, involving GABA_A_-mediated currents that are excitatory in neonatal neurons (Dzhala et al., 2005). Presumably, bumetanide reduces intracellular accumulation of chloride in neonatal neurons through its antagonism of NKCC1 on neurons. We do not view it as being inconsistent that bumetanide could mediate its antiepileptic effects in neonatal neurons through its effects on synaptic mechanisms related to the excitatory GABAergic currents neonatal neurons, but through nonsynaptic mechanisms via its effect on activity-driven changes in the EVF in adult brains. It might even be expected that loop diuretics would be less efficaciously antiepileptic in the neonate than in the adult brain, before the full development of astrocyte numbers and the structure of the extracellular space.

Bumetanide was not found to be effective in blocking some types of seizure models in rat hippocampal slices (Margineanu & Klitgaard, 2006), and in human tissue slices taken from patients with mesial temporal lobe epilepsy (Huberfeld et al., 2007). Bumetanide has very low solubility, and is a weak organic acid that partially dissociates into a negatively charged species in aqueous solution (Tata et al., 1993). Because of its physicochemical properties, bumetanide might not be expected to passively diffuse easily into tissue slices. Its lesser efficacy than furosemide in vitro may be due entirely to these properties. Indeed, in the study on human tissue (Huberfeld et al., 2007), low doses of bumetanide (5–10 μm) were used. Those doses were selected because they were found to antagonize NKCC1 in other studies on cultured cells (Payne, 1997). However, higher doses (e.g., in the millimolar range, as is required for furosemide), or other delivery vehicles for facilitating the penetration of bumetanide into tissue slices, might be required for effective blocking of activity-driven EVF changes in brain tissue slice experiments. In vivo, bumetanide and furosemide are bound (95% and 98%, respectively) to blood serum proteins, and hence possibly transported into the brain via organic anion transporters (OATs) (Kusuhara et al., 1999), similar to the mechanism by which they are transported into the kidney (Hasannejad et al., 2004). Alternatively, it may be that the small unbound portion of loop diuretics in the blood are efficiently distributed throughout the volume of the brain tissue via blood perfusion, and passive diffusion alone, across the blood–brain barrier and into the ECS, is sufficient to account for their greater ability to suppress epileptic activity at lower concentrations in vivo.

## Putting It All Together

We have discussed a series of results that support the notion that activity-driven changes of the EVF are necessary for the maintenance of hypersynchronous epileptiform activity in adult tissue. Different types of data provide support for transport of extracellular potassium and chloride are into glial cells via NKCC1 during neuronal activation, causing the swelling of glial cell processes through the generation of osmotic gradients. Treatments that block the activity-driven decreases in the EVF also block epileptiform activity regardless of the mix of synaptic and nonsynaptic mechanisms underlying the epileptic activity. These treatments include antagonism of NKCC1 with furosemide, bumetanide, low-[Cl^−^]_o_, or changing the osmolarity of the ECS. It appears that the action potentials between pairs of neurons become desynchronized with the blockade of activity-driven changes in the EVF. This mechanism is nonsynaptic, and appears to be independent of effects on action potential generation, neuronal excitability, or synaptic transmission. We suggest that the critical mechanism for the maintenance of hypersynchony is the dynamic activity-driven reduction of the EVF, rather than static changes in the EVF. This suggestion seems reasonable, given that low-[Cl^−^]_o_ and furosemide block the dynamic activity reduction in the EVF without significantly altering the baseline EVF at rest, as measured by ion selective microelectrodes (Holthoff & Witte, 1996).

The important question that remains unanswered is: why are the activity-driven changes in the EVF necessary for hypersynchony? Some possible answers are suggested by theoretical and computational models. An analysis suggested that the partitioning of the micro-local ECS by fine cellular processes creates the conditions for single action potentials, through a potassium-mediated self-excitation process, to evolve into an “autogenic” paroxysmal discharge. Similar suprathreshold ionic coupling between adjacent neuronal membranes can yield “cooperative” paroxysmal discharges through the same potassium-mediated mechanism (Lebovitz, 1996). This mechanism represents a potential synchronizing process that could be disrupted by treatments that affect the EVF. A computational study, incorporating currently available data, suggests that glial NKCC1 and Na+/HCO_3_− cotransporters are the only possible transporters that could account for the activity-driven reductions of the EVF that are observed experimentally (Østby et al., 2009). Together, these analyses suggest that the swelling and shrinking of glial process around neuronal cell bodies could facilitate hypersynchrony through a potassium-mediated process that depends on K+ transients at micro-local domains.
